# Oral Microbial Profile Analysis in Patients with Oral and Pharyngeal Cancer Reveals That Tumoral *Fusobacterium nucleatum* Promotes Oral Cancer Progression by Activating YAP

**DOI:** 10.3390/microorganisms11122957

**Published:** 2023-12-10

**Authors:** Yuki Yamamoto, Tomonori Kamiya, Megumu Yano, Vu Thuong Huyen, Masahiro Oishi, Miki Nishio, Akira Suzuki, Kishiko Sunami, Naoko Ohtani

**Affiliations:** 1 Department of Otolaryngology and Head & Neck Surgery, Graduate School of Medicine, Osaka Metropolitan University, Osaka 545-8585, Japan; 2 Department of Otolaryngology and Head & Neck Surgery, Graduate School of Medicine, Osaka City University, Osaka 545-8585, Japan; 3 Department of Pathophysiology, Graduate School of Medicine, Osaka Metropolitan University, Osaka 545-8585, Japan; 4 Division of Molecular and Cellular Biology, Kobe University Graduate School of Medicine, 7-5-1 Kusunoki-cho, Chuo-ku, Kobe 650-0017, Japan; 5 AMED-CREST, Japan Agency for Medical Research and Development (AMED), Tokyo 100-0004, Japan

**Keywords:** *Fusobacterium nucleatum*, oral and pharyngeal cancer, oral microbiome, Yes-associated protein (YAP)

## Abstract

The incidence of oral cancer has recently been increasing worldwide, particularly among young individuals and women. The primary risk factors for head and neck cancers, including oral and pharyngeal cancers, are smoking, alcohol consumption, poor oral hygiene, and repeated exposure to mechanical stimuli. However, approximately one-third of the patients with oral and pharyngeal cancers are neither smokers nor drinkers, which points to the existence of other mechanisms. Recently, human microbes have been linked to various diseases, including cancer. Oral pathogens, especially periodontal pathobionts, are reported to play a role in the development of colon and other types of cancer. In this study, we employed a series of bioinformatics analyses to pinpoint *Fusobacterium nucleatum* as the predominant oral bacterial species in oral and pharyngeal cancer tissue samples. We successfully isolated *Fn. polymorphum* from the saliva of patients with oral cancer and demonstrated that *Fn. polymorphum* indeed promoted oral squamous cell carcinoma development by activating YAP in a mouse tongue cancer model. Our research offers scientific evidence for the role of the oral microbiome in oral cancer progression and provides insights that would help in devising preventative strategies against oral cancer, potentially by altering oral bacterial profiles.

## 1. Introduction

Recently, between 350,000 and 400,000 new cases of oral cancer were reported annually worldwide, with over 170,000 fatalities resulting from the disease [1]. In Japan, approximately 6000 new cases are reported each year, leading to around 3000 deaths. The incidence is rising, notably among young individuals and women in Western countries. Oral cancer is categorized based on its location—the buccal mucosa, upper gingiva, lower gingiva, hard palate, tongue, and floor of the mouth. The tongue is the most frequent site for oral cancer occurrence followed by the gingiva, floor of the mouth, and buccal mucosa [2]. Histopathologically, over 90% of oral cancers are identified as squamous cell carcinomas (oral squamous cell carcinoma, OSCC). The remaining are other types of carcinomas or sarcomas originating from the salivary gland. Epidemiologically, this cancer is more prevalent in middle-aged and older men. The recognized risk factors include smoking, alcohol consumption, poor oral hygiene, and repeated mechanical stimuli, such as ill-fitting dentures [3,4]. However, about 33% of patients with oral cancer neither smoke nor drink alcohol, indicating the potential influence of other mechanisms [5,6,7,8].

In the human oral cavity, over 700 bacterial species, both commensal and heterologous, engage in intercellular communication and are pivotal in sustaining a balanced oral physiological environment [9,10]. Conversely, oral bacteria play roles in oral conditions like periodontal disease and dental caries and can impact the entire body by causing bacteremia and septicemia, including infective endocarditis [11]. Recent studies have highlighted that oral bacteria, such as periodontopathogens, including *Fusobacterium* species, are implicated in not only oral malignancies but also in distant cancers like colorectal, esophageal, and pancreatic cancers [12,13,14]. However, there is a limited body of research elucidating the mechanism by which the oral microbiota affects the progression of head and neck cancers, including oral and pharyngeal cancers.

To explore this, we examined the oral microbial compositions of saliva, dental plaque, tongue plaque, and fresh tumor biopsies from 65 patients diagnosed with oral, oropharyngeal, or hypopharyngeal cancers. These patients visited the Department of Otolaryngology and Head and Neck Surgery at Osaka Metropolitan University Hospital between April 2020 and August 2023 and fulfilled the inclusion criteria. Furthermore, we isolated oral bacteria from saliva samples of patients with oral and pharyngeal cancers and control individuals. The potential of these bacteria to induce tongue cancer was assessed using a mouse model.

## 2. Materials and Methods

### 2.1. Ethics

All experiments utilizing human samples were approved by the Ethics Committee of Osaka Metropolitan University (approval numbers: 2020-079 and 2021-282) in accordance with the Declaration of Helsinki. All patients and participants provided written informed consent to participate in the study. All the mouse experiments were approved by the Animal care and use Committee of Osaka Metropolitan University (approval number: 22012).

### 2.2. Patients

This study was conducted at the Department of Otolaryngology and Head and Neck Surgery, Osaka Metropolitan University Hospital, Osaka, Japan, between April 2020 and August 2023. This was a retrospective study using samples collected over the designated period. Sixty-five patients with oral and pharyngeal cancer (diagnosed as squamous cell carcinoma, SCC) and ninety-three control patients without oral cancer participated in the study and provided written informed consent. First, 125 head and neck cancer patients and 95 control patients with mild middle ear inflammation without head and neck cancers entered this study and provided written informed consent. After the application of the exclusion criteria, the number of patients with oral and pharyngeal cancer decreased to 65, and that of the control patients decreased to 93. The exclusion criteria were age < 20 years, a history of radiotherapy, antibiotic treatment, p16^INK4a^ positivity, indication of human papillomavirus (HPV) infection in the tumor area, tumors other than histologically squamous cell carcinoma (SCC), and head and neck cancers developed in the larynx. Patients’ clinical metadata are provided in the Supplementary Table S2.

### 2.3. Preparation and Storage of Oral Samples

Fresh biopsy samples of oral and pharyngeal cancer tissues, along with oral samples of saliva, dental plaque, and tongue plaque, were collected from patients within the criteria mentioned above. Samples were obtained during medical examinations at the Department of Otolaryngology and Head and Neck Surgery at Osaka Metropolitan University Hospital. Fresh biopsy samples (diameters 2–3 mm) were obtained using biopsy forceps at the first examination without prior rinsing of the mouth and were snap-frozen and used for 16S rRNA sequencing analysis. Approximately 2 mL of saliva was collected from each participant into a sterile tube. This sample was centrifuged at 100× *g* for 5 min at 4 °C to remove the contaminants. The supernatant was subsequently centrifuged at 5000× *g* for 10 min at 4 °C. The resulting pellet was either directly stored at −80 °C for 16S rRNA sequencing analysis or resuspended in Gifu anaerobic medium (GAM) modified with 20% glycerol and stored at −80 °C for bacterial stock. Tongue plaque samples were collected using a swab and immersed in 2 mL of PBS. This suspension was centrifuged at 5000× *g* for 10 min at 4 °C. The pellet was then either stored directly at −80 °C for 16S rRNA sequencing analysis or resuspended in GAM with 20% glycerol and stored at −80 °C for bacterial stock. Dental plaque samples were taken under anesthesia at the beginning of surgeries for oral and pharyngeal cancer or middle ear inflammation. These samples were divided into two portions: one was directly stored at −80 °C for 16S rRNA sequencing analysis, while the other was suspended in GAM medium supplemented with 20% glycerol and stored at −80 °C for bacterial stock. All samples were kept on ice prior to freezing.

### 2.4. DNA Extraction for Next-Generation Sequencing

Bacterial DNA was extracted from oral samples by BIKEN Biomics Inc. (Osaka, Japan) using an automated DNA extraction machine (GENE PREP STAR PI-480, Kurabo Industries Ltd., Osaka, Japan) and an NR-201 DNA extraction kit (Kurabo Industries Ltd., Osaka, Japan). The extraction was performed according to the manufacturer’s instructions. In brief, 0.5 g of glass beads (0.1 mm diameter, IEDA Trading Corp., Tokyo, Japan) and 300 μL of No. 10 solution (NR-10025) were added to each sample (200 μL each). This mixture was then agitated using a DISRUPTOR-GENIE (Scientific Industries Inc., Bohemia, NY, USA) at 3000 rpm for 90 s. Following this, the samples were centrifuged at 9700× *g* for 5 min. The supernatant was then collected and transferred to machine-compatible strips of eight sample tubes. Subsequently, 150 μL of No. 2 solution (NR-2025), supplemented with proteinase K (final concentration 0.4 mg/mL, FUJIFILM Wako Pure Chemical Corporation, Osaka, Japan), and another 150 μL of No. 10 solution (NR-10025) were added. The samples were then processed in the GENE PREP STAR PI-480 automated DNA extraction machine. The concentration of the extracted DNA was determined using a Qubit assay (Thermo Fisher Scientific Inc., Waltham, MA, USA). Extracted DNA samples were stored at −30 °C until further use.

### 2.5. 16S Ribosomal RNA (16S rRNA) Gene Amplification and Sequencing

DNA library preparation and sequencing were conducted by BIKEN Biomics Inc. (Osaka, Japan). Each DNA library was prepared following the Illumina 16S Metagenomic Sequencing Library Preparation Guide, using 27Fmod (5′-AGRGTTTGATCMTGGCTCAG-3′) and 338R (5′-TGCTGCCTCCCGTAGGAGT-3′) primers, which target the V1–V2 hypervariable region of the bacterial 16S rRNA gene. Samples were analyzed via 250 bp paired-end sequencing on a MiSeq system (Illumina, Inc. San Diego, CA USA) using the MiSeq Reagent v2 500 cycle kit (Illumina, Inc., San Diego, CA USA). All the bacterial sequence data are available in DDBJ BioProject. The accession number is PRJDB16847.

### 2.6. Oral Microbiota Profiling

Sequence reads were analyzed using the Quantitative Insights into Microbial Ecology 2 version 2021.11 (QIIME2, https://qiime2.org, accessed on 1 September 2023) pipeline. The specific QIIME2 commands and options are provided in Table S1. Acquired paired-end sequences were denoised and merged using the DADA2 R library within QIIME2. The taxonomic assignment of the 16S rRNA sequences was conducted using the Silva 138 99% OTU classifier. Amplicon sequence variants (ASVs) were aligned with the Mafft software in QIIME2 version 2021.11 and then subjected to diversity analysis using the fasttree software within QIIME2 version 2021.11. The observed ASVs, Shannon–Wiener indices, and Pielou’s evenness indices were determined at a sequence depth of 7321 reads per sample, undergoing 10 random iterations of the feature unit table via QIIME2. To maintain consistent sequencing depth, sample libraries were subsampled to 7321 reads for the Weighted Unifrac distance, representing the minimum number of reads across all libraries. β-Diversity and principal coordinates analysis (PCoA) plots were generated using the Weighted Unifrac distance in QIIME2. ASVs were analyzed using the National Center for Biotechnology Information (NCBI) BLASTN (http://blast.ncbi.nlm.nih.gov.gate1.inist.fr/ Blast.cgi, accessed on 1 September 2023) and the 16S RNA sequence database for species identification (identity > 97%, coverage > 95%). If the ASVs did not meet these criteria, the sequences were interpreted as representative of other species within the respective genus.

### 2.7. Shotgun Metagenomics Sequencing and Processing

For shotgun sequencing, 20 ng of the extracted DNA was sonicated to 300 bp using a Covaris ME220 instrument (Covaris, Inc., Woburn, MA, USA). Sonicated DNA was purified using a 1.0× volume of AMpure XP Beads (Beckman Coulter, Brea, CA, USA). Libraries were prepared using the KAPA Hyper Prep Kit (KAPA Biosystems, Wilmington, MA, USA) according to the manufacturer’s instructions. Paired-end (2 × 150 bp) sequencing was carried out on an Illumina NovaSeq 6000 (Illumina Inc., San Diego, CA, USA). Adapters and low-quality reads (*Q-score* < 25) were trimmed using fastp (v.0.23.2; https://github.com/OpenGene/fastp accessed on 1 January 2022). Reads shorter than 50 bp were discarded, and the first and last base sequences of the remaining reads were removed. Host-associated reads were filtered out using KneadData (v.0.10.0, http://huttenhower.sph.harvard.edu/kneaddata accessed on 1 January 2022) employing the default parameters and the hg37dec v0.1 reference. Functional annotation was executed using the Humann3 pipeline (v3.8; https://huttenhower.sph.harvard.edu/humann/ accessed on 1 September 2023). Sequences that were neither integrated nor mapped were excluded from the analysis. Pathways were identified by summing up the normalized counts per million.

### 2.8. Fluorescence In Situ Hybridization (FISH)

FISH was carried out on formalin-fixed, paraffin-embedded oral cancer tissue samples. Sections were hybridized with the 5′-Alexa Fluor 488-labeled universal bacterial probe EUB338 (5′-GCTGCCTCCCGTAGGAGT-3′) and the 5′-Texas Red-labeled *Fusobacterium nucleatum*-specific probe FUSO664 (5′-CTTGTAGTTCCGCYTACCTC-3′). Slides were deparaffinized and treated with wash buffer (0.9 M NaCl, 20 mM Tris-HCl (pH 7.5), and 0.1% SDS) for 60 min at 42 °C. They were then hybridized overnight with the specified FISH probes at a concentration of 2 μM in hybridization buffer (0.9 M NaCl, 20 mM Tris-HCl (pH 7.5), 0.01% SDS, and 20% formamide) at 42 °C. Post hybridization, slides were rinsed for 60 min at 42 °C in wash buffer. Tissue sections were then counterstained with DAPI and mounted. Images were captured using an IX71 fluorescence microscope (Olympus, Tokyo, Japan).

### 2.9. Detection of Fusobacterium nucleatum Subspecies and Isolation of F. nucleatum Strain

To detect the *Fusobacrterium nucleatum* subspecies, 1 ng of saliva DNA sample was used for each reaction. The PCR conditions were 35 cycles of 98 °C for 30 s, 55 °C for 30 s, and 72 °C for 20 s using the KOD One^®^ PCR Master Mix (TOYOBO, Osaka, Japan) on an S1000TM thermal cycler (Bio-Rad, Hercules, CA, USA) according to the manufacturer’s protocol. The following primers were used for the detection of each *F. nucleatum* subspecies as described previously [15]. *Fn nucleatum*; SR-n3 N3-F 5′-CAAGCAACTGAAAATGCTTTAAAAG-3′ and N3-R 5′-TCCAGGTAAGGAAATTACACCTACTG-3′. *Fn animalis*; SR-a2 A2-F 5′-ACTCAAATTATTATGAATGTGATGAAAGA-3′ and A2-R 5′-GCTACTGAAGGATGAAATGCTGG-3′. *Fn vincentii*; SR-v1 V1-F 5′-GAGGCTATTGCAAATTAAACTGTTAAA-3′ and V1-R 5′-CTTTACCACTATTATAAACTAAATAAATGAGAC-3′. *Fn polymorphum* SR-p3 P3-F 5′-CYTATGGYTTTGATTTTGACTTATTTG-3′ and P3-R 5′-CCAAAGTAATTAAAGCCTCTTGAGC-3′.

To isolate *F. nucleatum* strains, bacterial stock from the oral cavity samples of oral cancer patients was seeded onto modified FM agar plates (Nissui, Tokyo, Japan). The cultures were then incubated under anaerobic conditions (85% N_2_, 5% H_2_, 10% CO_2_) in an anaerobic chamber (Baker Ruskinn, Sanford, ME, USA) for 2–4 days at 37 °C. For species identification, the 16S rRNA gene was amplified from selected individual colonies using bacterial universal PCR primers (Bact-27Fmod: 5′-AGRGTTTGATYMTGGCTCAG-3′, Bact-1492R: 5′-GGYTACCTTGTTACGACTT-3′). PCR was carried out with the KOD One^®^ PCR Master Mix (TOYOBO, Osaka, Japan) on an S1000TM thermal cycler (Bio-Rad, Hercules, CA, USA) following the manufacturer’s protocol. Amplification was confirmed using gel electrophoresis on 1.0% agarose gels. PCR products were then purified with the FastGene™ Gel/PCR Extraction kit (NIPPON Genetics Co, Ltd., Tokyo, Japan) according to the manufacturer’s instructions. The sequencing of the 16S rRNA gene from the bacterial PCR products was conducted using the BigDye Terminator v3.1 Cycle Sequencing Kit (Applied Biosystems, Carlsbad, CA, USA) and sequencing primers (Bact-27F, Bact-1492R). The DNA was purified through ethanol precipitation and sequenced on ABI 3130x1 capillary sequencers (Applied Biosystems). The resultant sequences were analyzed with the NCBI (National Center for Biotechnology Information) BLASTN and the 16S RNA sequence database to ascertain species identification (identity > 97%, coverage > 95%).

### 2.10. Conditional Mob1a/b DKO (YAP-Activated) Mice as a Tongue Cancer Model

The generation of Mob1a/b homozygous double-mutant mice (Rosa26-CreERT;Mob1a^flox/flox^; Mob1b^−/−^) has been previously reported [16]. To excise the floxed *Mob1a* gene, 15 μL of tamoxifen (Sigma-Aldrich) diluted in 100% ethanol (10 mg/mL) was applied directly and gently to the mouse tongue daily for 5 days using a small soft brush, starting at 4 weeks of age. Prior to tamoxifen administration, mice were anesthetized using pentobarbital and isoflurane. From 5 weeks of age, mice were given water containing the antibiotics, 1.6 mg/mL sulfamethoxazole and 0.32 mg/mL trimethoprim, for 5 days. To prepare the *F. nucleatum* inoculum, the isolated strain was cultured in modified Gifu anaerobic medium (GAM) broth (Nissui, Tokyo, Japan) to confluence for 1 day at 37 °C. The cultured medium underwent centrifugation at 3000× *g* for 15 min at room temperature. The resultant pellet was carefully washed twice with phosphate-buffered saline (PBS) to remove medium components. Subsequently, the pellet was resuspended in 0.5 mL PBS containing 2% carboxymethyl cellulose and applied to the mice’s tongues (approximately 1 × 10^9^ CFU in 50 μL medium per mouse).

### 2.11. Immunostaining

Tongue tissues from mice were fixed in 4% paraformaldehyde (PFA), embedded in paraffin, and sectioned (5 μm) following standard procedures. For antibody staining, sections that were deparaffinized and rehydrated underwent heat-induced antigen retrieval for 20 min using Retrieval Solution (S1699, Dako, Santa Clara, CA, USA). After a PBS wash, the sections were treated with a blocking reagent (HK085-5K, BioGenex, San Ramon, CA, USA) for 15 min at room temperature. They were then incubated with primary antibodies targeting Ki67 (RM-9106-S0, Thermo Fisher Scientific Inc.) and YAP1 (#4912S, Cell Signaling Technology Inc., Beverly, MA, USA) overnight at 4 °C. After another PBS wash, the sections were exposed to either a peroxidase-conjugated secondary antibody (MK202, Takara Bio Inc. Kusatsu Shiga, Japan) or an Alexa Fluor 594-conjugated secondary antibody (A-21207, Invitrogen) for 1 h at room temperature. Certain slides were developed with nickel-enhanced 3,3′-diaminobenzidine (DAB, MK210, Takara Bio Inc. Kusatsu Shiga, Japan) for anti-Ki67 and counterstained with Mayer’s Hematoxylin and 4′,6-diamidino-2-phenylindole (DAPI, 19178-91, Nacalai, Kyoto, Japan) for immunofluorescence staining. They were then mounted using either Entellan™ new (1.07961.0100, Sigma-Aldrich Pty Ltd, an affiliate of Merck KGaA, Darmstadt, Germany) or ProLong™ Gold Antifade Mountant (P36930, Thermo Fisher Scientific Inc., Waltham, MA, USA ). The stained sections were visualized and captured using a fluorescence microscope (BZ-X800; Keyence, Osaka, Japan), with analysis conducted using BZ-X software version 1.1.1.8 (BZ-H4A, Keyence, Osaka, Japan). For Ki67-positivity assessments, the entire tongue epithelial area of each mouse was measured using BZ-X software version 1.1.1.8, and the proportion of the Ki67-positive region was determined. Similarly, the area with YAP1-positive nuclear staining, co-stained with DAPI, was evaluated with BZ-X software version 1.1.1.8, and the fraction of the nuclear YAP1-positive region within the entire tongue epithelial area was computed.

### 2.12. Statistical Analysis

Statistical analyses were performed using GraphPad Prism software version 8.4.3 (GraphPad Software Inc., La Jolla, CA, USA) and R software (version 4.0.3). A heatmap correlating to samples based on the relative abundance of genus-level taxa was generated using Ward’s method and hierarchical clustering with the pheatmap package v1.0.12 in R software (see Supplementary Figure S2). Data were analyzed using the unpaired *t*-test, the chi-square test, Fisher’s exact test, the Kruskal–Wallis test, and the pairwise Wilcoxon rank-sum test with the Benjamini–Hochberg adjustment, as specified in the text or figure legends. Spearman’s correlation coefficient assessed the correlation between groups. For all tests, a *p*-value of <0.05 was considered statistically significant.

## 3. Results

### 3.1. Bioinformatics Analysis of the Oral and Tumor Microbial Profiles in Patients with Oral and Pharyngeal Cancers Using 16S rRNA Gene Amplicon Sequencing Data

**Figure 1:**
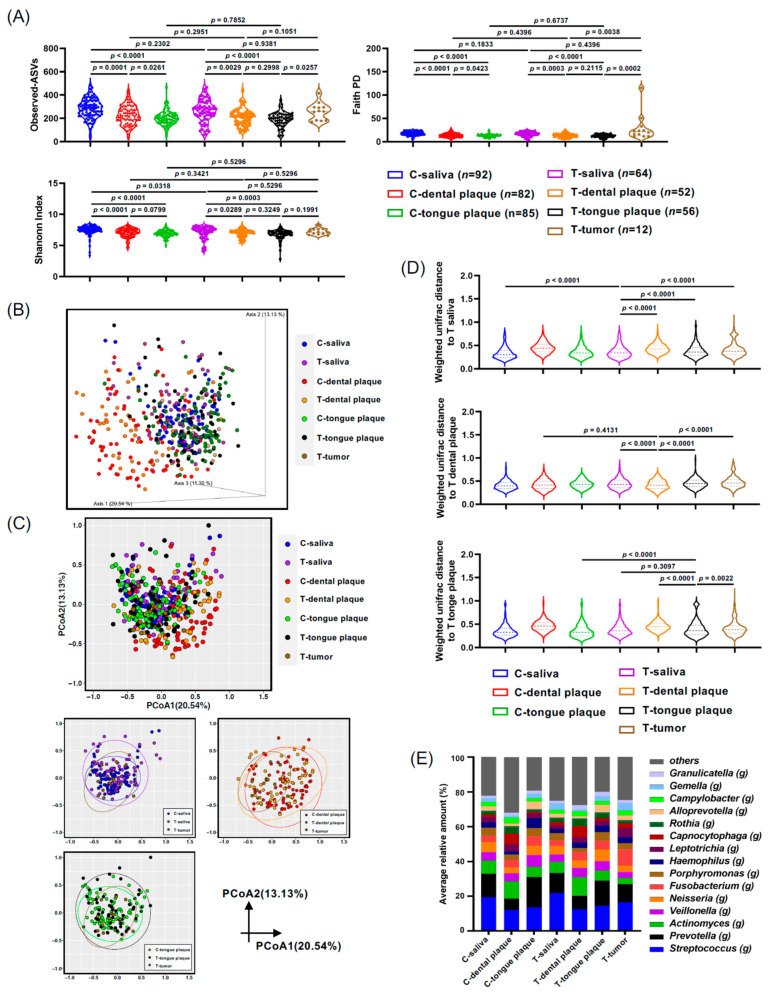
Microbiota from the saliva, dental plaque, tongue plaque, and tumor tissue of the oral cancer (T-) and control groups (C-). (**A**) Violin plots depicting the alpha diversity using the Shannon index and Faith PD. (**B**,**C**) Weighted UniFrac principal coordinates analysis (PCoA) of oral microbiota compared between the oral cancer and control groups (3D plot (**B**) and 2D plot (**C**)). (**D**) Violin plots displaying the Weighted UniFrac distances to saliva (T-saliva), dental plaque (T-dental plaque), and tongue plaque (T-tongue plaque) in oral cancer. (**E**) Oral microbiota composition determined through 16S rRNA gene sequencing. The genus-level taxonomic distribution of individual microbes is displayed. Data in (**A**,**D**) were compared between the two indicated groups using a pairwise Wilcoxon rank-sum test with a Benjamini–Hochberg adjustment.

As described in Section 2 and Table 1, saliva, dental plaque, and tongue plaque samples were collected from controls and patients with oral and pharyngeal cancers. Tumor biopsy samples were also collected from patients with oral and pharyngeal cancer, and bacterial profiles were analyzed. The Shannon Index and Faith PD analysis of the control saliva profile exhibited significant differences when compared to the dental (*p* < 0.0001) and tongue plaque (*p* < 0.0001) samples of the controls (Figure 1A). Additionally, significant disparities between the bacterial profiles in the saliva of cancer patients and their dental samples (*p* = 0.0289 and *p* = 0.0003 in Shannon Index and Faith PD analysis, respectively) or tongue plaque samples (*p* = 0.0003 and *p* < 0.0001 in Shannon Index and Faith PD analysis, respectively) were detected (Figure 1A). Moreover, α-diversity displayed a marked distinction between the bacterial communities in tumor patients’ tongue plaque and tumor tissue biopsy as per the Faith PD analysis (*p* = 0.0002, Figure 1A). The oral cancers diagnosed in this study were identified as squamous cell carcinoma (OSCC) (Table 1). Henceforth, samples from controls or patients with oral and pharyngeal cancers are denoted as C-saliva or T-saliva, C-dental plaque or T-dental plaque, and C-tongue plaque or T-tongue plaque, signifying saliva, dental plaque, or tongue plaque samples from control (C-) individuals or those with oral and pharyngeal cancers (T-).

The β-diversity analysis, illuminating microbial composition differences between various groups, overall showed similarities in bacterial distributions across the saliva, tongue plaque, and tumor tissues as visualized in the three-dimensional plots of PCoA computed via Weighted UniFrac distance (Figure 1B). Nevertheless, the bacterial distribution in C-dental plaque, represented as red dots, and in T-dental plaque, represented as yellow dots, displayed distinct patterns when compared to those of T-saliva, T-tongue plaque, or T-tumor tissue. However, no statistical difference was observed between these dental plaque samples from controls and those from cancer patients (C-dental plaque and T-dental plaque), suggesting a likeness in their bacterial profiles, likely due to the anaerobic microenvironment (Figure 1C). Notably, the T-tumor samples exhibited significant dissimilarity from T-saliva, T-dental plaque, and T-tongue plaque when assessed using the weighted UniFrac distance analysis (Figure 1D). Bacterial profiles at the genus level for all samples are depicted in Figure 1E, further emphasizing the apparent contrast in bacterial compositions in dental plaque when juxtaposed with other oral samples (Figure 1E).

### 3.2. Oral Cancer-Associated Bacteria in Non-Drinkers and Non-Smokers

**Figure 2:**
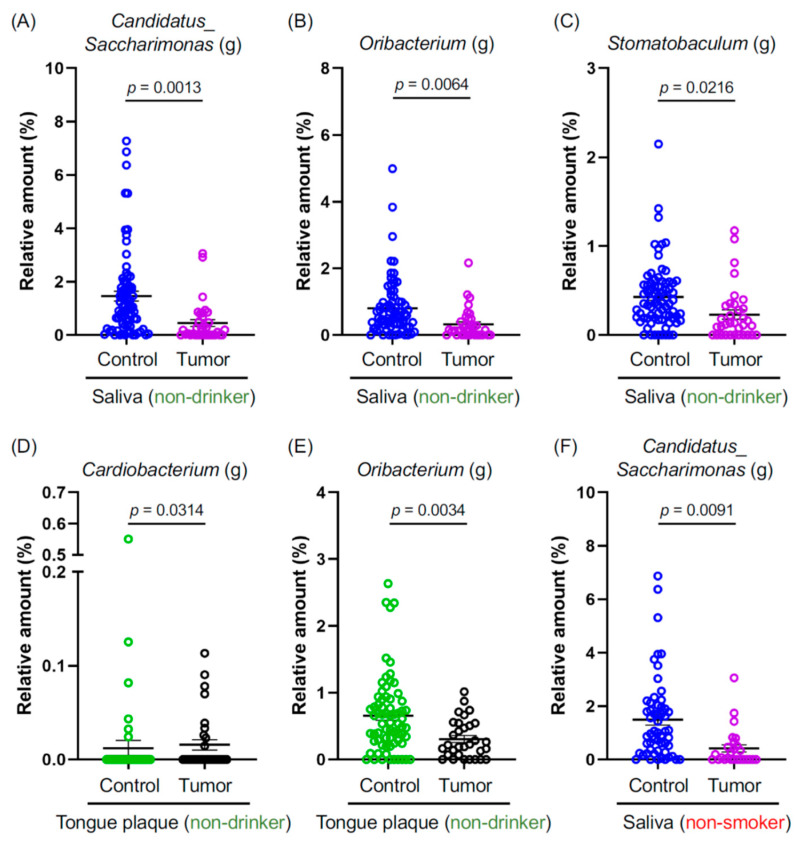
(**A**–**F**) Graphs depict the relative abundance at the genus level, where genera showed significant differences between the indicated groups. Data are presented as mean ± SEM, compared between the two groups using a pairwise Wilcoxon rank-sum test with Benjamini–Hochberg adjustment. Blue: The saliva of controls. Purple: The saliva of cancer patients. Green: The tongue plaque of controls. Black: The tongue plaque of cancer patients.

Smoking and alcohol consumption are well-established carcinogenic agents that promote oral cancer [17]. They also induce dysbiosis in the oral cavity [18,19]. However, the prevalence of oral cancer in non-smokers and non-drinkers has been on the rise [6,7]. Considering this, we probed whether specific oral microbiota are either increased or decreased in the tumor tissues of non-smokers and non-drinkers. Our sample comprised 53.8% (35 out of 65 HNSCC patients) of non-drinking and 40.0% (26 out of 65 HNSCC patients) of non-smoking patients with oral and pharyngeal cancers (Table 1). Genus-level bacteria, namely *Candidatus Saccharimonas* (*g*), *Oribacterium* (*g*), and *Stomatobaculum* (*g*), were significantly reduced in the comparison between C- and T-saliva samples (Figure 2A–C). *Cardiobacterium* (*g*) and *Oribacterium* (*g*) exhibited a significant decrease among non-drinkers when compared between C- and T-tongue plaque samples (Figure 2D,E). Intriguingly, *C. Saccharimonas* (*g*) also showed a notable reduction in non-smokers with tumors (Figure 2F). In summary, the aforementioned oral bacterial species were significantly diminished in patients with oral and pharyngeal cancers who are non-drinkers and non-smokers, suggesting that these bacterial species might offer protective effects against HNSCC and play a role in preserving oral health. 

### 3.3. Bacterial Species Associated with Oral and Pharyngeal Cancer Development

To identify bacterial species contributing to oral and pharyngeal cancer development, we examined oral bacteria, which increased significantly in samples from patients with oral and pharyngeal cancer using multiple comparison tests. Bacteria that exhibited significant increases or decreases are illustrated in Supplementary Figure S1. At the genus level, *C. Saccharimonas* (*g*), *Leptotrichia* (*g*), *Megasphaera* (*g*), *Stomatobaculum* (*g*), and *Oribacterium* (*g*) were significantly less abundant in T-saliva samples compared to C-saliva samples (Supplementary Figure S1A). However, no significantly increased bacterial species were detected in T-saliva. In our analysis of bacterial species in C- and T-tongue plaques, *Capnocytophaga* (*g*) and *Actinobacillus* (*g*) were slightly but significantly increased in the T-tongue plaque samples (Supplementary Figure S1B). No significant alterations in bacteria were observed when comparing C-dental to T-dental plaque samples.

**Figure 3:**
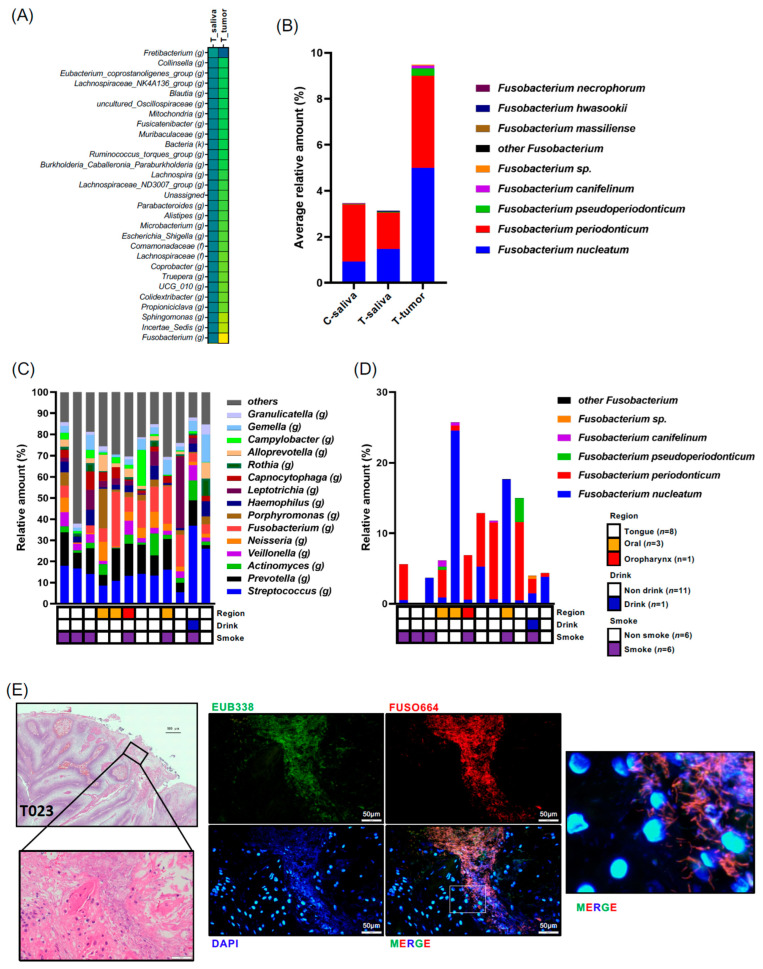
(**A**) Differences in microbiota composition at the genus level are illustrated using the scaling method of unit variance between saliva and tumor from patients with oral cancer. Highlighted genera were significant at an adjusted *p*-value < 0.05. Data in (**A**) were compared between the indicated groups using a pairwise Wilcoxon rank-sum test with Benjamini–Hochberg adjustment. (**B**) Composition of the Fusobacterium genus is displayed. (**C**) Tumor microbiota composition determined through 16S rRNA gene sequencing for each individual patient. The genus-level taxonomic distribution of individual microbes is displayed. (**D**) Composition of the Fusobacterium genus is displayed for each individual patient. (**E**) HE staining and FISH analysis with the EUB338 probe for all bacteria (green) and FUSO664 for Fusobacterium nucleatum (red) in the tumor region of oral cancer patients (No. T023). Nuclei were stained with DAPI (blue). Scale bar: 20 μm.

Subsequently, our focus shifted to bacteria enriched in patients’ oral tumor tissues. We compared bacterial species between each sample and tumor tissue samples, with significantly altered bacteria shown in Supplementary Figure S1C–I. Several bacteria were identified as significantly increased or decreased in tumor tissue samples. Notably, *Fusobacterium* (*g*) exhibited a significant increase in T-tumor samples compared to T-saliva samples with the highest fold change (fold change = 3.021) (Figure 3A, Supplementary Figure S1C). Species-level analysis highlighted that within the *Fusobacterium* genus, *F. nucleatum* was the most prevalent in tumor tissue, followed by *F. periodontium* (Figure 3B). Next, we investigated the individual cancer microbial profiles and *Fusobacterium* species in the biopsy samples (*n* = 12) (Figure 3C). However, the tendency of site-specific bacterial profile was not observed, partly because of the limited number of samples. We found that not only *Fusobacterium nucleatum* but also *Fusobacterium periodonticum* were abundant in the cancer tissues (Figure 3D). Moreover, *F. nucleatum* was indeed detected in the patients OSCC tissues via FISH analysis using *Fn*-specific probes (Figure 3E). Given that the *Fusobacterium* genus is predominant in tumor tissues, we further explored the bacterial community coexisting with *Fusobacterium*. Relationships among the tumoral bacteria and the top 50 bacterial genera in the tumor tissue samples were analyzed using Spearman’s correlations and visualized in a heatmap using the pheatmap package in R software (v1.0.12) for correlations with r values between 10.5 and 20.5 (Supplementary Figure S2). Predominantly, robust positive bacterial communities were observed, including *Veillonella* (*g*), *Stomatobaculum* (*g*), *Megasphaera* (*g*), and *Oribacterium* (*g*), and another group containing *Bacteroides* (*g*), *Kingella* (*g*), *Faecalibacterium* (*g*), and *Blautia* (*g*) in tumor tissue samples (Supplementary Figure S2). Specifically regarding the *Fusobacterium* genus, there were several significantly negative co-occurrences with bacteria such as *Rothia* (*g*) and *Lactobacillales* (*o*) (*p*-values; *p* = 0.0105 and *p* = 0.0126, respectively, and correlation coefficient R-values; r = −0.69231 and r = −0.70474, respectively) (Supplementary Figure S2). However, no strongly positive co-occurring bacteria with *Fusobacterium* were observed, suggesting that *Fusobacterium* tends to exist in tumor tissue without specific bacterial community ties.

The further analysis of bacteria positively or inversely correlated with *Fusobacterium* (*g*) was conducted using Spearman’s rank correlation coefficient for both control and tumor patient groups. *Veillonella* (*g*) and *Streptococcus* (*g*) emerged as inversely correlated bacteria across all samples (Supplementary Figure S3), aligning with the co-occurrence analysis findings (Supplementary Figure S2). Additionally, a shotgun metagenome analysis revealed that several nutrition- or energy-production-associated bacterial pathways were significantly increased in T tumor samples (Supplementary Figure S4).

### 3.4. Fusobacterium nucleatum from Patients with Oral Cancer Promotes OSCC in Mice

*Fusobacterium nucleatum* is a renowned oncogenic bacterium associated with colon, pancreatic, esophageal, oral, and other cancers [20,21]. Consequently, we sought to determine whether *F. nucleatum* indeed has a propensity to promote oral cancer using a mouse model.

Initially, we aimed to isolate bacterial species from the saliva samples of patients with oral cancer. *Fusobacterium nucleatum* encompasses several subspecies, including *Fn. nucleatum*, *Fn. polymorphum*, *Fn. vincentii*, *Fn. animalis*, *Fn. fusiforme*, and *Fn. canifelium*. Sequencing analysis identified the isolated *Fn* as *Fn polymorphum* from the saliva of a patient with oral cancer (Supplementary Figure S5). *Fusobacterium nucleatum polymorphum* is reported as the most enriched subspecies in oral cancer biopsies [22]. Our saliva samples from patients with oral cancer were then investigated to identify which subspecies were dominant. We checked the four major subspecies and found that our samples also included *Fn. polymorphum* with the highest occupancy (77.3% among the 52 patients samples examined) (Supplementary Figure S6). *Fn. vincentii* was also as dominant as *Fn. polymorphum* in our samples. While several reports indicate that *F. nucleatum* is present within oral cancer tissues [20,23], the direct oncogenic role of this bacterial species in oral cancer has remained undetermined. Thus, we employed this subspecies, *Fn. polymorphum*, to examine tongue cancer development in mice.

**Figure 4:**
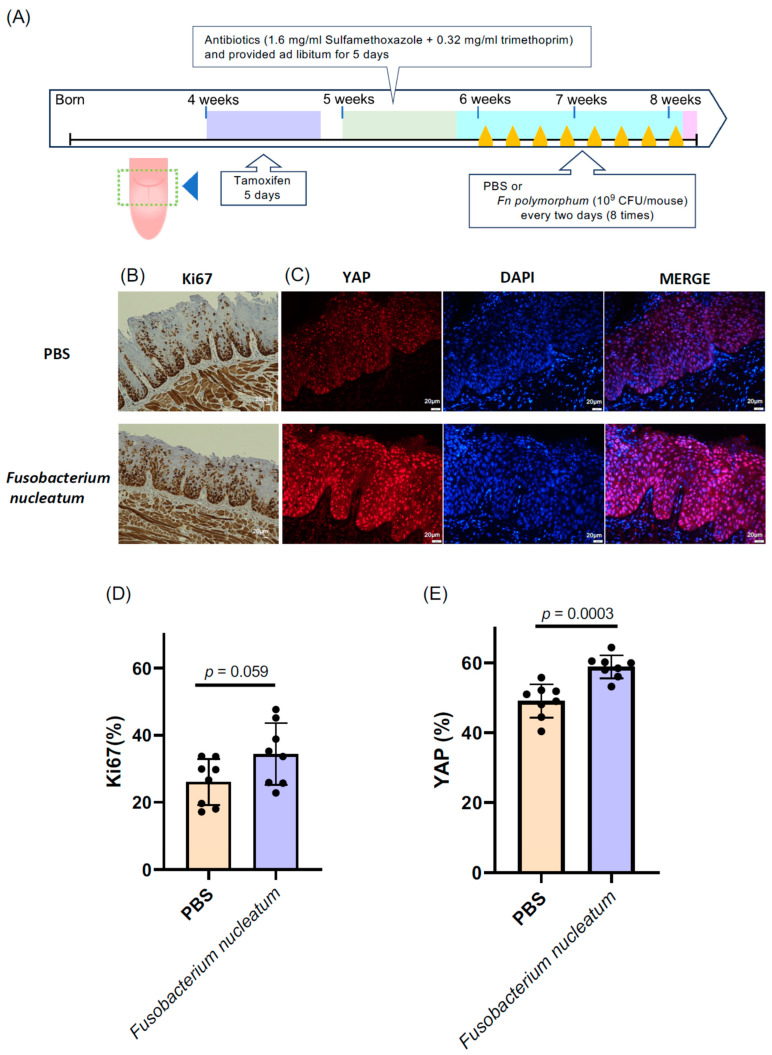
*Fusobacterium nucleatum* isolated from the oral cavity of oral cancer patients was applied to the tongues of epithelial cell-specific Mob1a/b DKO mice (tgMob1DKO). (**A**) Experimental scheme for *F. nucleatum*-treated tgMob1DKO mice. Tamoxifen was brushed daily for 5 days onto the tongues of 4-week-old tgMob1DKO mice. The application area of tamoxifen is illustrated in the dotted square. Mice received water containing antibiotics, sulfamethoxazole, and trimethoprim, for 5 days starting from 5 weeks old. Subsequently, mice were orally treated eight times with *F. nucleatum polymorphum* (*n* = 8) or PBS (*n* = 8) every two days post antibiotic administration. Mice were euthanized post treatment, and their tongue tissues were analyzed histologically. (**B**) Representative Ki67 immunostaining of tongue epithelium from mice in (**A**). Scale bar: 20 μm. (**C**) Representative images showcasing the immunofluorescent detection of YAP (red) in tongue epithelium from mice in (**A**). DAPI (blue) stains nuclei. Scale bar: 20 μm. (**D**) Percentages of Ki67-positive cells from sections in (**B**) are shown in bar plots. (**E**) Percentages of YAP-positive cells from sections in (**C**) are represented in bar plots. Data are presented as means ± SEM with Student’s t-test applied.

To delve into the role of *Fn. polymorphum* in oral cancer, we utilized a tongue cancer mouse model of tgMob1DKO mice, as described in Section 2 [16]. This genetically modified mouse strain develops tongue cancer upon tamoxifen exposure through the Cre-mediated excision of the floxed Mob1a gene. We administered 15 μL of a 10 mg/mL tamoxifen solution—less than the amount specified in the original report [16]—directly, yet gently, to a designated area of the mouse tongues (Figure 4A) daily for five days, inducing a mild epithelial hyperproliferation without aggressive OSCC. This subtle epithelial hyperproliferation is apt for exploring the influence of oral bacteria on OSCC progression.

Subsequently, after the tamoxifen treatment, we introduced *Fn. polymorphum* isolated from a patient with oral cancer onto the mice tongues. The proliferative activity was evaluated by determining the ratio of Ki67-positive cells and the expansion in the nuclear YAP localization area within the mouse tongue epithelium compared to that within the tamoxifen-treated control mouse tongues without bacterial treatment. Intriguingly, mice treated with *Fn. polymorphum* exhibited a significantly higher number of Ki67-positive cells across the tongue (Figure 4B). Additionally, the proportion of nuclear YAP-positive cells also increased significantly in *Fn. polymorphum*-treated mouse tongues (Figure 4C). These findings suggest that *Fn. polymorphum* sourced from a patient with OSCC may stimulate the oncogenic proliferation of oral epithelial cells. 

## 4. Discussion

The number of patients with head and neck cancers, including oral and pharyngeal cancers, has recently shown an increasing trend [24]. The major risk factors for oral cancers are smoking and alcohol consumption [3,4]. However, approximately one-third of patients with oral or pharyngeal cancers neither smoke nor drink alcohol, which indicates that other mechanisms could be involved in the development of oral cancer [5,6,7,8]. Therefore, as one of the potential mechanisms of oral and pharyngeal cancer development, we focused on oral microbiota.

This study explored the bacterial profile in 65 patients with oral and pharyngeal cancers by utilizing saliva, dental plaque, and tongue plaque samples, as well as tumor biopsies. The 65 cases of oral and pharyngeal cancers analyzed in this study included 35 non-drinkers and 26 non-smokers (Table 1). The abundance of several bacteria, such as *Candidatus Saccharimonas* (*g*), *Oribacterium* (*g*), and *Stomatobaculum* (*g*), was found to be significantly decreased in samples from non-drinkers and non-smokers who developed oral and pharyngeal cancers (Figure 2), which suggests that these oral microbes could be protective against oral cancer.

In addition, we identified *F. nucleatum* as the predominant oral bacterial species in tumor tissue samples, in contrast to the bacteria found in saliva samples from patients with oral cancer. We isolated *Fn. polymorphum* from the sample of patients with oral cancer and demonstrated its role in promoting oral cancer development using a mouse tongue cancer model.

*Fusobacterium nucleatum* is an anaerobic bacterium that belongs to the Gram-negative group. Typically found in the mouth, as well as in the urogenital tract and intestine, it usually behaves as an opportunistic pathogen. However, it is frequently detected in colorectal, oral, pancreatic, esophageal, and other cancers [12,13,14,20]. *F. nucleatum* is renowned for promoting tumors via the activation of the Wnt/β-catenin pathway [20,25]. Moreover, *F. nucleatum* can modulate antitumor immunity by affecting the antitumor activities of NK cells and cytotoxic T cells through Fap2, thereby inhibiting T-cell activity via TiGIT [26].

Several subspecies of *F. nucleatum* have been identified, including *Fn. nucleatum*, *Fn. polymorphum*, *Fn. vincentii*, *Fn. animalis*, *Fn. fusiforme*, and *Fn. canifelium*. Prior research indicated that *F. nucleatum polymorphum* was the most significantly enriched species in oral cancer biopsies from 20 cases [22]. Multiple studies have confirmed the presence of *F. nucleatum* within oral cancer tissues [20,27], but the capacity of these bacteria to induce oral cancer has not been thoroughly explored. We also confirmed that *F. nucleatum polymorphum* was the most abundant subspecies in our saliva samples from patients with oral and pharyngeal cancers. Our findings reveal that patient-derived *F. nucleatum polymorphum* promotes oral epithelial proliferation and increases the proportion of nuclear YAP-positive cells. YAP activation is reported to be vital for the progression of oral cancer [16,28]. Therefore, the YAP activation signaling underscores the potential of *F. nucleatum polymorphum* to intensify oral cancer severity.

Another study focused on the role of intratumoral bacteria in human oral cancer tissues [27]. This research delved into stage-specific bacteria and prognostically relevant oral bacteria, such as *Capnocytophaga* (*g*). We also detected a slight yet significant increase in *Capnocytophaga* (*g*) levels in T-tongue plaque samples compared with that in C-tongue plaque samples (Supplementary Figure S1B). Nonetheless, since *F. nucleatum* is more abundantly detected in oral cancer tissues, our study primarily centered on the influence of *F. nucleatum* on oral cancer evolution. This paper highlights alterations in stage-specific immune cell states, emphasizing CD4- and CD8-positive T cells and associated cytokines. Recently, it was reported that *F. nucleatum* produces short-chain fatty acids [29], which are known to activate Treg cells [30] and alter tissue immunity [29], reinforcing the theory that metabolites derived from *F. nucleatum* modulate anticancer immunity.

Our results could provide a tool for predicting the risk of oral and pharyngeal cancer using saliva samples. We identified *F. nucleatum polymorphum* as an oral cancer facilitator, the increase of which can be used to predict the risk of oral cancer. Moreover, the bacterial species, which showed reduced abundance in the saliva samples from patients with oral and pharyngeal cancer who were non-drinkers and non-smokers (Figure 2), can also be used for assessing the risk. These bacteria may also function in the prevention of oral and pharyngeal cancer and might be used as probiotics after further investigations.

This study has some limitations. The molecular mechanisms of how *F. nucleatum polymorphum* promoted the YAP activity, which might lead to the development of therapeutic drugs for oral cancer has not been clarified. Also, the cancer-promoting potential of other *F. nucleatum* subspecies remains to be elucidated in vivo using animal experiments. We also did not examine the landscape of tumor microenvironment in oral and pharyngeal cancer tissues including the infiltration of immune cells in the tumor. Oral microbiota produces a variety of metabolites that could affect the function of immune cells, which we would like to investigate further. For this, we need to increase the number of tumor samples so that the intratumoral bacteria can be examined.

In conclusion, we demonstrate that *F. nucleatum* is the most prevalent oral bacterial species in tumor tissue samples from patients with oral and pharyngeal cancers via a series of bioinformatics analyses. After isolating *Fn. polymorphum* from patients with oral and pharyngeal cancers, we confirmed its role in facilitating oral cancer progression in a mouse model. We also delved into bacteria that either co-occur with *F. nucleatum* or exhibit significant negative correlations. The results offer compelling evidence for the role of oral bacteria in the pathogenesis of oral and pharyngeal cancers and provide new insights into potential preventative strategies against oral cancer, perhaps by adjusting the composition of oral bacteria.

## Figures and Tables

**Table 1:** Summary of patients investigated in this study. HNSSC in this study includes oral, oropharyngeal, and hypopharyngeal cancers.

	Total		Male		Female	
	HNSCC * n = 65 (24/7/34)	Control *n* = 93	HNSCC * n = 44 (12/3/29)	Control *n* = 47	HNSCC * n = 21 (12/4/5)	Control *n* = 46
Age (average)	68.6	55.3	67.0	53.0	72.0	58.0
(range)	(33–89)	(24–84)	(33–85)	(24–80)	(45–89)	(28–84)
Clinical Stage						
(I/II/III/IV)	28/18/7/12		21/11/4/8		7/7/3/4	
Drinking						
non-drinkers	35 (20/5/10 *)	77	21 (10/3/8 *)	36	14 (10/2/2 *)	41
current	30 (4/2/24 *)	16	23 (2/0/21 *)	11	7 (2/2/3 *)	5
Smoking						
non-smokers	26 (13/2/11 *)	57	13 (4/0/9 *)	24	13 (9/2/2 *)	33
current	39 (11/5/23 *)	36	31 (8/3/20 *)	23	8 (3/2/3 *)	13

*: The number of patients with oral/oropharynx/hypopharynx cancers. Oral cancer consists of tongue (male *n* = 7; female *n* = 7), oral-floor (male *n* = 1; female *n* = 1), gingival (male *n* = 2; female *n* = 4), and buccal mucosal (male *n* = 2; female *n* = 0) cancers.

## Data Availability

The datasets presented in this study are available in the DDBJ database.

## Supplementary Materials

The following supporting information can be downloaded at: https://www.mdpi.com/article/10.3390/microorganisms11122957/s1. Figure S1: Comparison of microbial composition in the samples of the oral cavity; Figure S2: Co-occurrence analysis of bacteria in tumor tissues from oral cancer patients; Figure S3: Correlation between the abundance of *Fusobacterium* (*g*) and other bacteria; Figure S4: Metagenomic analysis of oral bacteria; Figure S5: Isolated *Fusobacterium* was identical to *Fn polymorphum*; Figure S6: The occupancy of Fusobacterium subspecies in patients’ saliva samples; Table S1: Analysis workflow and QIIME2 parameter settings; Table S2: Sample metadata.
