# Supplementary material for: Oral Microbial Profile Analysis in Patients with Oral and Pharyngeal Cancer Reveals That Tumoral Fusobacterium nucleatum Promotes Oral Cancer Progression by Activating YAP
Source: Microorganisms. 2023 Dec 10;11(12):2957. doi: 10.3390/microorganisms11122957 (PMC10746018; doi:10.3390/microorganisms11122957)
Supplement: Supplementary file 1 [file microorganisms-11-02957-s001.zip › Supplementary Figures 1 to 6 20231209.pdf]

# Supplemental Figure. S1

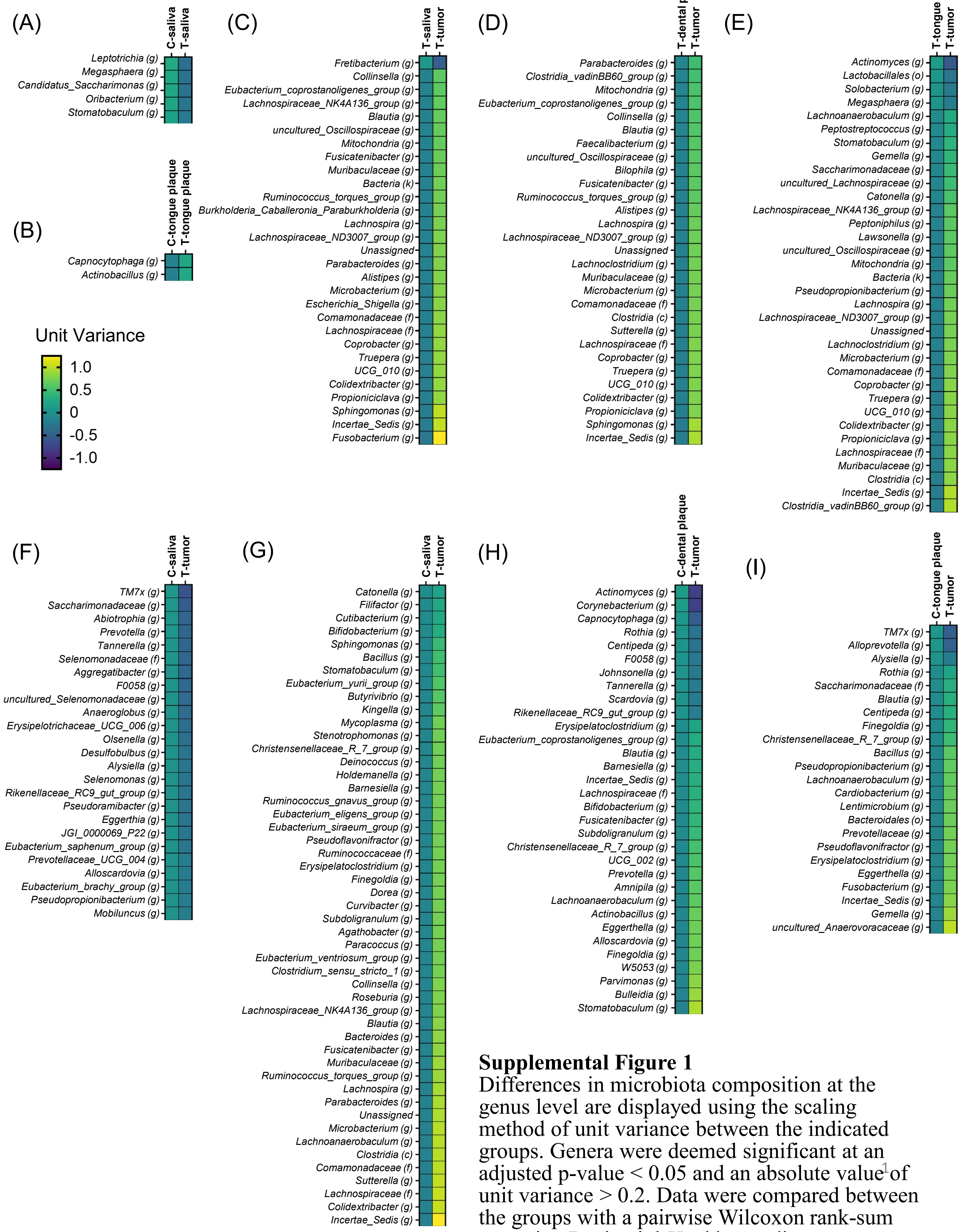


**Supplemental Figure 1**
Differences in microbiota composition at the genus level are displayed using the scaling method of unit variance between the indicated groups. Genera were deemed significant at an adjusted p-value < 0.05 and an absolute value of unit variance > 0.2. Data were compared between the groups with a pairwise Wilcoxon rank-sum test using Benjamini-Hochberg adjustment.

# Supplemental Figure. S2

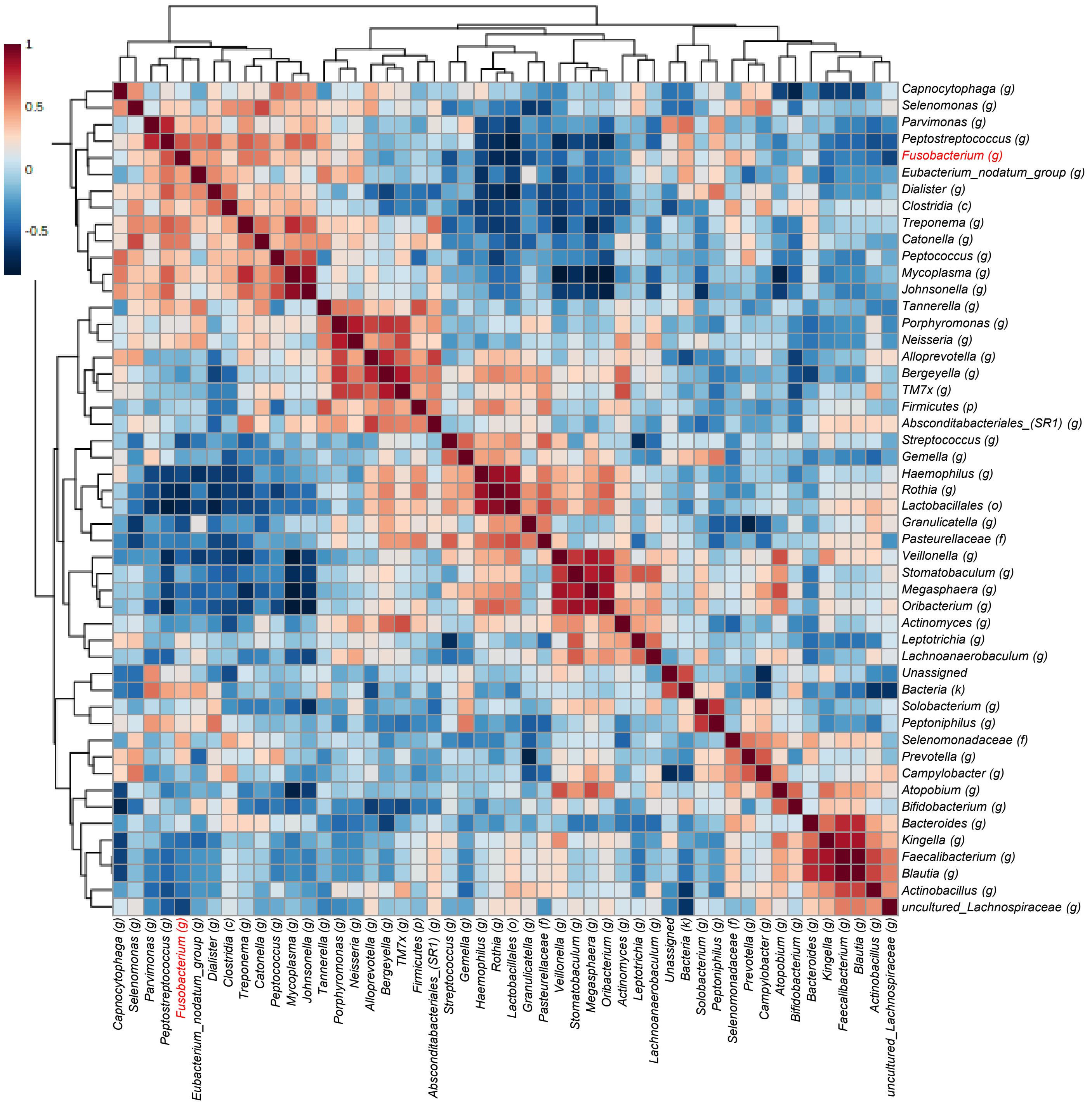


**Supplemental Figure 2**
Co-occurrence analysis of the top 50 bacterial genera in tumor tissues from oral cancer patients is depicted using Spearman's rank correlation coefficient in a heat map.

Supplemental Figure. S3

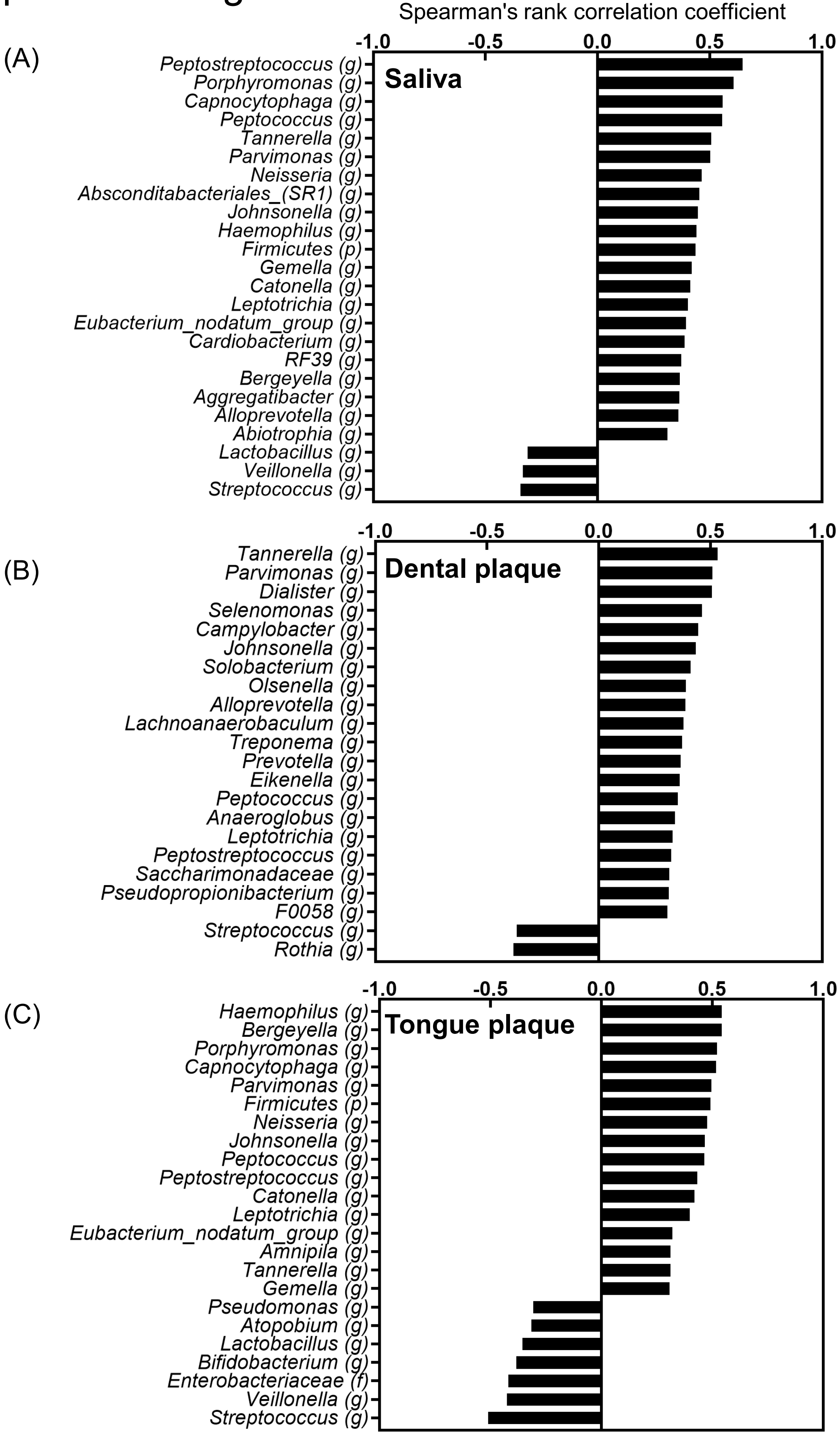




**Supplemental Figure 3**

Correlation between the abundance of Fusobacterium and other bacterial genera in saliva (A), dental plaque (B), and tongue plaque (C) between the control and oral cancer patient groups is showcased using Spearman's rank correlation coefficient in bar plots.

# Supplemental Figure. S4

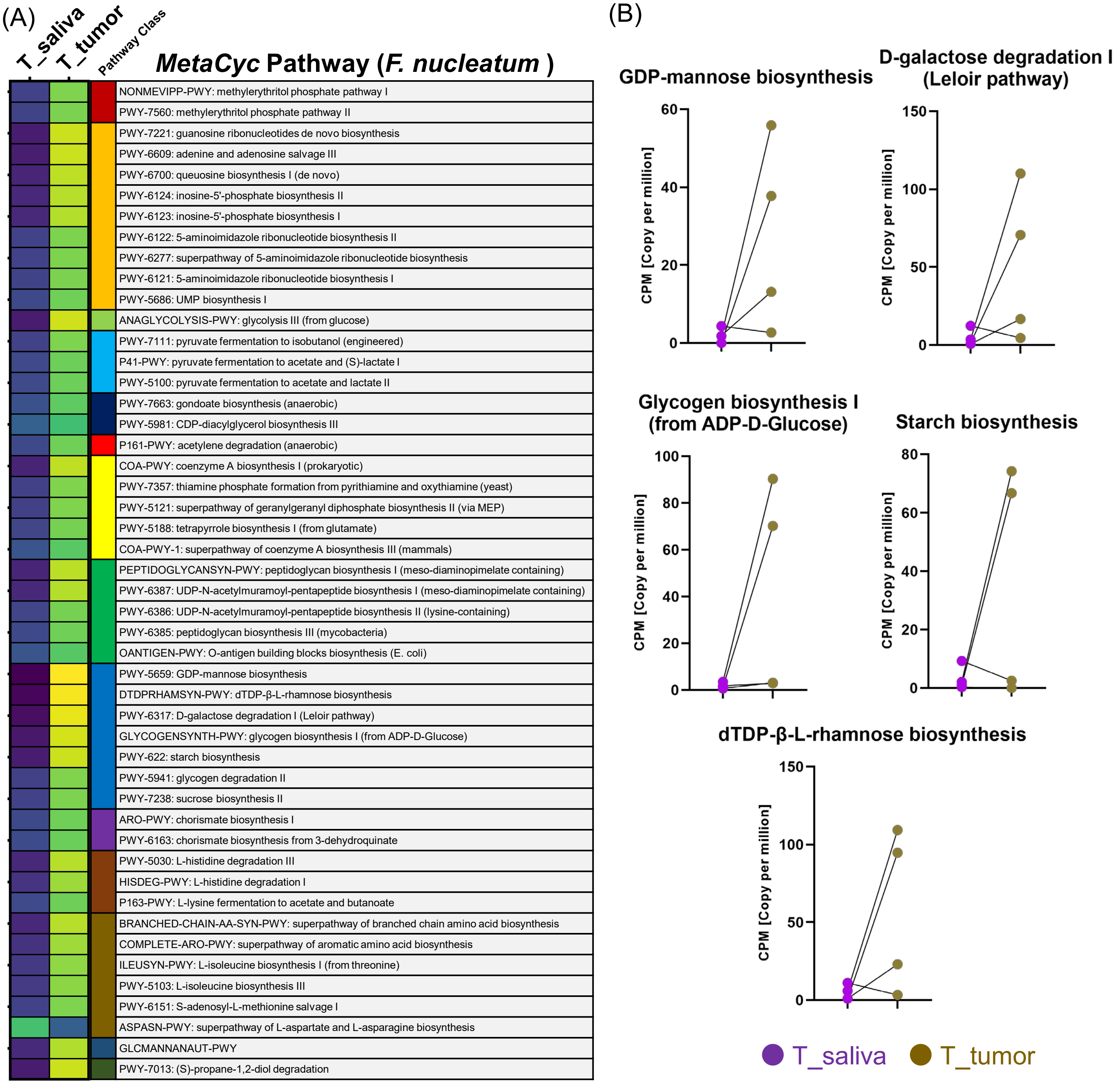


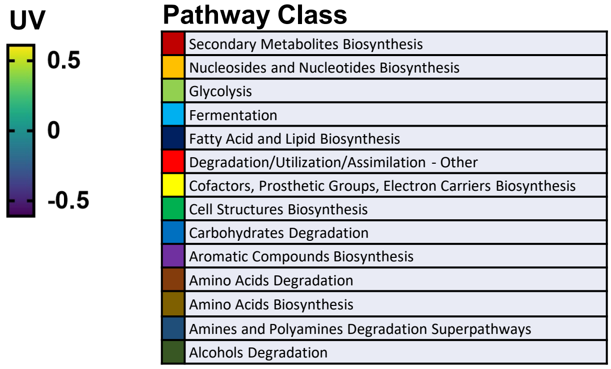


**Supplemental Figure 4**
(A) All microbial pathways of *Fusobacterium nucleatum* associated with saliva and tumors from oral cancer patients were identified with the HUMAnN3 tool, analyzing microbial gene families. Pathway abundances are based on copies per million (CPM), and differences in CPM are displayed using the scaling method of unit variance between the groups. (B) Indicated pathway abundances of *F. nucleatum* in saliva and tumors from the same oral cancer patients are shown.

# Supplemental Figure. S5

**Fusobacterium nucleatum subsp. polymorphum strain KCOM 1260 (=ChDC F218) genome**

Sequence ID: CP021934.1 Length: 2635767 Number of Matches: 5

Range 1: 1706311 to 1707158 GenBank Graphics ▼ Next Match ▲ Previous Match

| Score | Expect | Identities | Gaps | Strand |
|---|---|---|---|---|
| 1554 bits(841) | 0.0 | 847/850(99%) | 2/850(0%) | Plus/Minus |

```
Query  1        TCCTAGGATTATGAAAGCTATATGCGCTGTGAGAGAGCTTTGCGTCCCATTAGCTAGTTG  60
                ||||||||||||||||||||||||||||||||||||||||||||||||||||||||||||
Sbjct  1707158  TCCTAGGATTATGAAAGCTATATGCGCTGTGAGAGAGCTTTGCGTCCCATTAGCTAGTTG  1707099

Query  61       GAGAGGTAACGGCTCACCAAGGCGATGATGGGTAGCCGGCCTGAGAGGGTGATCGGCCAC  120
                ||||||||||||||||||||||||||||||||||||||||||||||||||||||||||||
Sbjct  1707098  GAGAGGTAACGGCTCACCAAGGCGATGATGGGTAGCCGGCCTGAGAGGGTGATCGGCCAC  1707039

Query  121      AAGGGGACTGAGACACGGCCCTTACTCCTACGGGAGGCAGCAGTGGGGAATATTGGACAA  180
                ||||||||||||||||||||||||||||||||||||||||||||||||||||||||||||
Sbjct  1707038  AAGGGGACTGAGACACGGCCCTTACTCCTACGGGAGGCAGCAGTGGGGAATATTGGACAA  1706979

Query  181      TGGACCAAGAGTCTGATCCAGCAATTCTGTGTGCACGATGAAGTTTTTCGGAATGTAAAG  240
                ||||||||||||||||||||||||||||||||||||||||||||||||||||||||||||
Sbjct  1706978  TGGACCAAGAGTCTGATCCAGCAATTCTGTGTGCACGATGAAGTTTTTCGGAATGTAAAG  1706919

Query  241      TGCTTTCAGTTGGGAAGaaaaaaaTGACGGTACCAACAGAAGAAGTGACGGCTAAATACG  300
                ||||||||||||||||||||||||||||||||||||||||||||||||||||||||||||
Sbjct  1706918  TGCTTTCAGTTGGGAAGAAAAAAATGACGGTACCAACAGAAGAAGTGACGGCTAAATACG  1706859

Query  301      TGCCAGCAGCCGCGGTAATACGTATGTCACAAGCGTTATCCGGATTTATTGGGCGTAAAG  360
                ||||||||||||||||||||||||||||||||||||||||||||||||||||||||||||
Sbjct  1706858  TGCCAGCAGCCGCGGTAATACGTATGTCACAAGCGTTATCCGGATTTATTGGGCGTAAAG  1706799

Query  361      CGCGTCTAGGTGGTTATGTAAGTCTGATGTGAAAATGCAGGGCTCAACTCTGTATTGCGT  420
                ||||||||||||||||||||||||||||||||||||||||||||||||||||||||||||
Sbjct  1706798  CGCGTCTAGGTGGTTATGTAAGTCTGATGTGAAAATGCAGGGCTCAACTCTGTATTGCGT  1706739

Query  421      TGGAAACTGTGTAACTAGAGTACTGGAGAGGTAAGCGGAACTACAAGTGTAGAGGTGAAA  480
                ||||||||||||||||||||||||||||||||||||||||||||||||||||||||||||
Sbjct  1706738  TGGAAACTGTGTAACTAGAGTACTGGAGAGGTAAGCGGAACTACAAGTGTAGAGGTGAAA  1706679

Query  481      TTCGTAGATATTTGTAGGAATGCCGATGGGGAAGCCAGCTTACTGGACAGATACTGACGC  540
                ||||||||||||||||||||||||||||||||||||||||||||||||||||||||||||
Sbjct  1706678  TTCGTAGATATTTGTAGGAATGCCGATGGGGAAGCCAGCTTACTGGACAGATACTGACGC  1706619

Query  541      TGAAGCGCGAAAGCGTGGGTAGCAAACAGGATTAGATACCCTGGTAGTCCACGCCGTAAA  600
                ||||||||||||||||||||||||||||||||||||||||||||||||||||||||||||
Sbjct  1706618  TGAAGCGCGAAAGCGTGGGTAGCAAACAGGATTAGATACCCTGGTAGTCCACGCCGTAAA  1706559

Query  601      CGATGATTACTAGGTGTTGGGGGTCGAACCTCAGCGCCCAAGCAAACGCGATAAGTAATC  660
                ||||||||||||||||||||||||||||||||||||||||||||||||||||||||||||
Sbjct  1706558  CGATGATTACTAGGTGTTGGGGGTCGAACCTCAGCGCCCAAGCAAACGCGATAAGTAATC  1706499

Query  661      CGCCTGGGGAGTACGTACGCAAGTATGAAACTCAAAGGAATTGACGGGGACCCGCACAAG  720
                ||||||||||||||||||||||||||||||||||||||||||||||||||||||||||||
Sbjct  1706498  CGCCTGGGGAGTACGTACGCAAGTATGAAACTCAAAGGAATTGACGGGGACCCGCACAAG  1706439

Query  721      CGGTGGAGCATGTGGTTTAATTCGACGCAACGCGAGGAACCTTACCAGCGTTTGACATCT  780
                ||||||||||||||||||||||||||||||||||||||||||||||||||||||||||||
Sbjct  1706438  CGGTGGAGCATGTGGTTTAATTCGACGCAACGCGAGGAACCTTACCAGCGTTTGACATCT  1706379

Query  781      TAGGAATGAGACAGANATGTTTCAGTGTCCCTTCGGGGGAAACCTAAAGACAGGTGGTGC  840
                ||||||||||||||| |||||||||||||||||||||| |||||||||||||||||||||
Sbjct  1706378  TAGGAATGAGACAGAGATGTTTCAGTGTCCCTTCGGGG-AAACCTAAAGACAGGTGGTGC  1706320

Query  841      ATGGGCTGTC  850
                |||| |||||
Sbjct  1706319  ATGG-CTGTC  1706311
```

**Supplemental Figure 5**

Isolated *Fn* from oral cancer patient’s saliva was 99% identical to *Fn polymorphum* by BLAST search.

# Supplemental Figure. S6

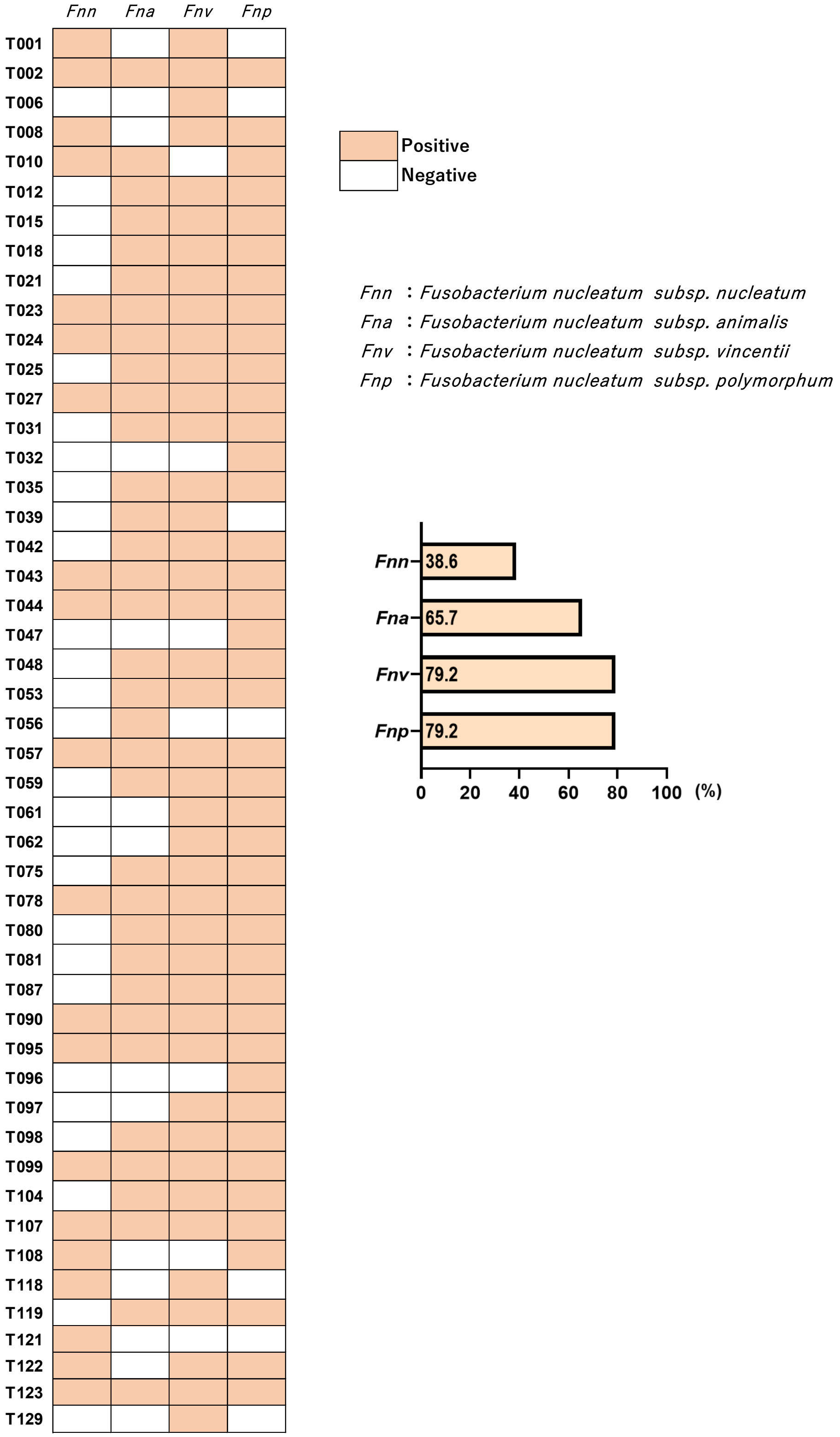


**Supplemental Figure 6**
The existence of four major *Fn* subspecies in oral cancer patients' saliva is displayed. Orange highlighted squares show the existence of *Fn* subspecies as indicated.
